# *DJ-1*: A Potential Biomarker Related to Prognosis, Chemoresistance, and Expression of Microenvironmental Chemokine in HR-Positive Breast Cancer

**DOI:** 10.1155/2023/5041223

**Published:** 2023-12-13

**Authors:** Yinghong Xie, Yuancheng Li, Mengzhu Yang

**Affiliations:** ^1^Department of Pathology, The First Affiliated Hospital of Soochow University, Suzhou 215006, Jiangsu, China; ^2^Institute of Dermatology, Jiangsu Key Laboratory of Molecular Biology for Skin Diseases and STIs, Chinese Academy of Medical Sciences and Peking Union Medical College, Nanjing 210042, Jiangsu, China; ^3^Department of Geriatric Oncology, The First Affiliated Hospital of Nanjing Medical University, Nanjing 210029, Jiangsu, China; ^4^Core Facility Center, The First Affiliated Hospital of Nanjing Medical University, 300 Guangzhou Road, Nanjing 210029, Jiangsu, China

## Abstract

*DJ-1* is significantly elevated in various malignancies. However, the clinical significance of *DJ-1* in hormone receptor (HR)-positive (HR+) breast cancer remains unclear. We evaluated *DJ-1* expression in different databases and validated *in vitro* assay by RT-PCR and western blot among HR+ breast cancer. The correlations between *DJ-1* level and tumor-immune were calculated. Mutational landscape, enriched signaling pathways, and drug sensitivity analyses were also assessed between *DJ-1* high and low-expression groups. *DJ-1* was upregulated in HR+ breast cancer, and high *DJ-1* expression was significantly linked with poor prognosis. *DJ-1* was correlated with the expression and function of different immune cells. The low *DJ-1* group showed sensitivity to paclitaxel and docetaxel, while the high-expression group showed sensitivity to doxorubicin. CTLA4 and PD-L1 were more sensitive in high-*DJ-1* group. It is involved in a range of pathways and might behave as a novel biomarker of prognostic value for the immune environment and drug sensitivity in HR+ breast cancer.

## 1. Introduction

Globally, breast cancer is the most common cancer in women and ranked the most common cause of cancer-related mortality among women [1]. It is known to all that the hormone receptor (HR) status, such as estrogen receptor (ER) and progesterone receptor (PR), play important roles in prognostic and treatment in breast cancer [2]. Tumor subtypes with expression of either ER or PR in at least 1% of the biopsied tumor cells are categorized as HR-positive (HR+) subtypes [3]. This subtype accounts for the majority of all breast cancers, approximately 65%–70%, and causes most of the victims from this disease [4, 5]. The most important molecule underlying the HR+/HER2− subtype is ER*α*, a steroid HR and a transcription factor. When ER*α* is activated by estrogen, it could activate oncogenic growth pathways in breast cancer cells. Although endocrine therapy that blocks the ER pathway has been developed for years and shows great effectiveness [6], more dysregulated molecules that may serve as novel treatment targets need to be identified.

*DJ-1*, known as one member of the peptidase C56 family, was originally known for its protective role against oxidative stress and cell death in Parkinsonism [7, 8]. Beyond that, *DJ-1* has been reported in cancers. The evidence shows that *DJ-1* may be involved in various mechanisms in cancer progression, including the inhibition of cellular apoptosis, redox sensing, acting as a marker for chemotherapy resistance, suppression of ferroptosis, regulating histone glycation, and inhibition of autophagy [9–13]. It has been identified overexpression in a range of cancer types, including breast cancer [14, 15], osteosarcoma [16], melanoma [17], colorectal cancer [18], endometrial cancer [19], and esophageal cancer [20], indicating its role as oncogene. Previously, a study in breast cancer cell lines has shown that NRG-I promotes the decoupling of DJ-I with HER3 and activates the heterodimerization of HER2/HER3 [21]. Scumaci et al. [22] reported that phospho-*DJ-1* can prevent glycation-induced histone dysregulation, and its Akt-related hyperactivity sustains the proliferation of cancer cells by preserving the epigenome landscape. Nowadays, immunotherapy therapy has become a promising strategy for breast cancer [23, 24], and *DJ-1* might exert a specific influence on immune cells. For instance, Treg homeostasis can be maintained via pyruvate dehydrogenase activity promoted by *DJ-1* [25], and the loss of *DJ-1* can reduce the number of total CD4+ T cells while increasing fractional thymic and peripheral nTregs [26]. *DJ-1* can also act as an immune modulator through regulating the activation of several immune cells, such as macrophages, mast cells (MCs), and T cells, via reactive oxygen species (ROS)-dependent and/or ROS-independent mechanisms [27]. However, the clinical significance and role in the immune environment of *DJ-1* among HR+ subtypes still remains unclear. Thus, to evaluate the value of the *DJ-1* in HR+ breast cancer is fundamental.

To help elucidate the possible relationship between the *DJ-1* expression and HR+ patient clinical features, immune environment, and chemosensitivity, we explored *DJ-1* both *in silico* and *in vitro*. Our results shed light on the importance of *DJ-1* in HR+ breast cancer as well as providing potential relationships and mechanisms between *DJ-1* and HR+ breast cancer immunotherapy.

## 2. Materials and Methods

### 2.1. Study Population

A total of 940 patients, including 591 HR+ /HER2− subtypes from The Cancer Genome Atlas (TCGA) breast cancer cohort, were included into our analysis. The hormone-based subtypes were inferred from immunohistochemistry results in the dataset. Their matched gene expression matrix (version 2017-10-13), clinic information, follow-up records, protein expression, and somatic mutation were also obtained from the UCSC Xena hub (https://xenabrowser.net/).

Another cohort containing 1,885 primary breast cancer patients with follow-up time and gene expression profiles, including 1,459 HR+/HER2− subtypes, from the Molecular Taxonomy of Breast Cancer International Consortium (METABRIC) project, was downloaded (https://www.mercuriolab.umassmed.edu/metabric) and utilized as external validation cohort for *DJ-1* expression and survival analysis.

We also used the publicly available datasets in the Oncomine database (https://www.oncomine.org/resource/main.html) [28] to verify the *DJ-1* expression between tumor and normal breast cancer tissues.

### 2.2. Survival Analysis of DJ-1

The correlation between *DJ-1* expression and breast cancer survival status was analyzed by grouping HR+ subtype patients into high and low *DJ-1* expression groups according to the median expression of *DJ-1*. Overall survival (OS) and progression-free interval (PFI)/relapse-free survival (RFS) were used as endpoints. We used the Cox regression model in the R *survminer* package (v0.4.9) to calculate and visualize the HR and Cox *P* values. We adjusted common confounding factors, age, and tumor stage as covariates during the regression.

### 2.3. Pathway Enrichment Analysis

To compare the biofunction difference between high and low *DJ-1* expression groups in the pathway level, we performed gene set variation analysis (GSVA) analysis. We first downloaded classic cancer hallmark pathways from the MSigDB Collections (https://www.gsea-msigdb.org/) and calculated GSVA pathway scores for each sample in the TCGA and METABRIC cohort by the *gsva* R package (v1.48.1). We then compared pathway scores between *DJ-1* high and low-expression groups by the *limma* package.

### 2.4. Somatic Mutation Analysis

We compared significant somatic mutation genes between high and low *DJ-1* expression groups in TCGA patients. The *maftools* package (v2.16.0) was used to calculate the tumor mutation burden (TMB) and generate the genomic profile diagram.

### 2.5. Immune Infiltration Analysis

We performed single-sample GSEA analysis on expression profiles to evaluate the phenotypes of classic human infiltrating immune cells [29]. The relationship between deduced immune cell fractions and *DJ-1* expression was analyzed by Spearman correlation.

### 2.6. Drug Sensitivity Analysis

We used the R package *pRRophetic* to assess the sensitivity of chemotherapeutic sensitivity for HR+ breast cancer patients by estimation of IC_50_ (half maximal inhibitory concentration). The pRRophetic algorithm is based on the pharmacogenomics database of Cancer Genome Project cell line data and the Cancer Cell Line Encyclopedia [30]. Generally, patients with high IC_50_ values are less sensitive to the tested drug. We compared deduced IC_50_ values from chemotherapeutic agents approved by the FDA between high and low *DJ-1* groups. Also, we compared the expression of target therapy-related biomarkers between groups such as TMB, PD1, and CTLA4.

### 2.7. In Vivo Validation of DJ-1 Expression

All the cell lines (MCF-10A, MCF-7, T-47D, SK-BR-3, BT-474, MDA-MB-231, and MDA-MB-468) were obtained from ATCC. BT-474 and T-47D were maintained in RPMI 1640 medium with 10% fetal bovine serum (FBS) and 1% penicillin/streptomycin. MDA-MB-468 was maintained in a Leibovitz's-15 (L-15) supplemented with with 10% FBS and 1% penicillin/streptomycin. The human MCF-10A mammary nontumorigenic epithelial cells were cultured in Dulbecco's modified eagle medium (DMEM)/F12 medium (3 : 1) supplemented with 10% horse serum, 0.5 *μ*g/ml hydrocortisone, 20 ng/ml recombinant epidermal growth factor, 10 *μ*g/ml insulin and antibiotics. MCF-7, SK-BR-3, and MDA-MB-231 were cultured in a DMEM medium with FBS, penicillin, and streptomycin. All cells were maintained in a humidified atmosphere containing 5% CO_2_ and 95% air at 37°C.

The total RNA of whole-cell lysates was isolated using Trizol reagent (Invitrogen, California, USA) according to the manufacturer's protocol and used in converting to cDNA with a First-Strand Synthesis System for RT-PCR (Nuo Weizan, China) according to the manufacturer's instructions. Quantitative real-time PCR was performed with a Roche LightCycler 96 Real-Time PCR System (Roche, Basel, Switzerland). The primers are listed in *Supplementary 1*.

All protein samples were isolated from cell lines, and cell samples were lysed in RIPA lysis buffer (Beyotime, China) supplemented with the protease inhibitor (Roche). Protein concentration was measured using a bicinchoninic acid protein assay kit (Thermo, USA). Proteins (30 *μ*g) were subjected to sodium dodecyl sulfate–polyacrylamide gel electrophoresis separation on 10% gels (Bio-Rad Laboratories, Hercules, CA), and proteins were transferred to a nitrocellulose membrane. The membrane was blocked with 5% milk powder for 1 hr before incubation with primary antibodies (*DJ-1*: Cell Signaling Technology, USA; Tubulin: Proteintech, USA) and horseradish peroxidase-conjugated secondary antibody. All western blot images were captured and quantified by enhanced chemiluminescent reagent (Thermo, USA).

The immunohistochemistry images of *DJ-1* protein were downloaded from the human protein atlas through the *Hpar* packages in R.

### 2.8. Statistical Analysis

All statistical tests were performed using the Wilcoxon rank-sum test for continuous data and the Spearman's rank correlation for the estimation of correlation. The Fisher's exact test was used for categorical data comparison. All statistical analysis was performed in R software (v4.1.3). Two-sided *P* values < 0.05 were considered statistically significant.

## 3. Results

### 3.1. DJ-1 Expression in Breast Cancer

**Figure 1:**
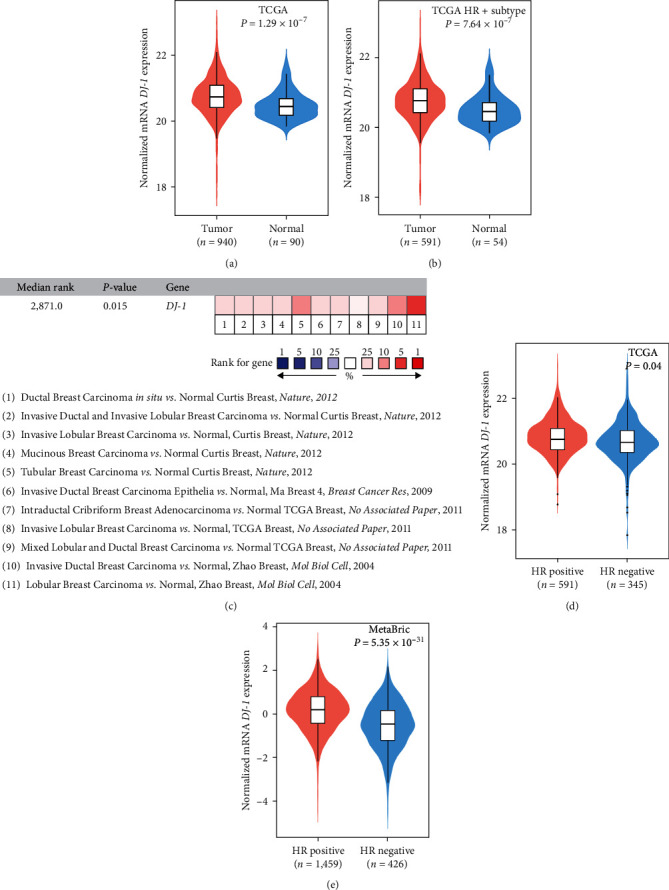
*DJ-1* expression in breast cancer: (a) differential expression of *DJ-1* between breast cancer and normal samples in TCGA breast cancer dataset; (b) differential expression of *DJ-1* between HR+ subtypes tumor and normal samples in TCGA dataset; (c) meta-analysis of *DJ-1* expression in Oncomine database with criteria as *P* < 0.05, log_2_ fold change ≥1 and top 10% gene rank; (d) and (e) expression of *DJ-1* between HR+ and HR− breast cancer in TCGA breast cancer and METABRIC dataset.

**Figure 2:**
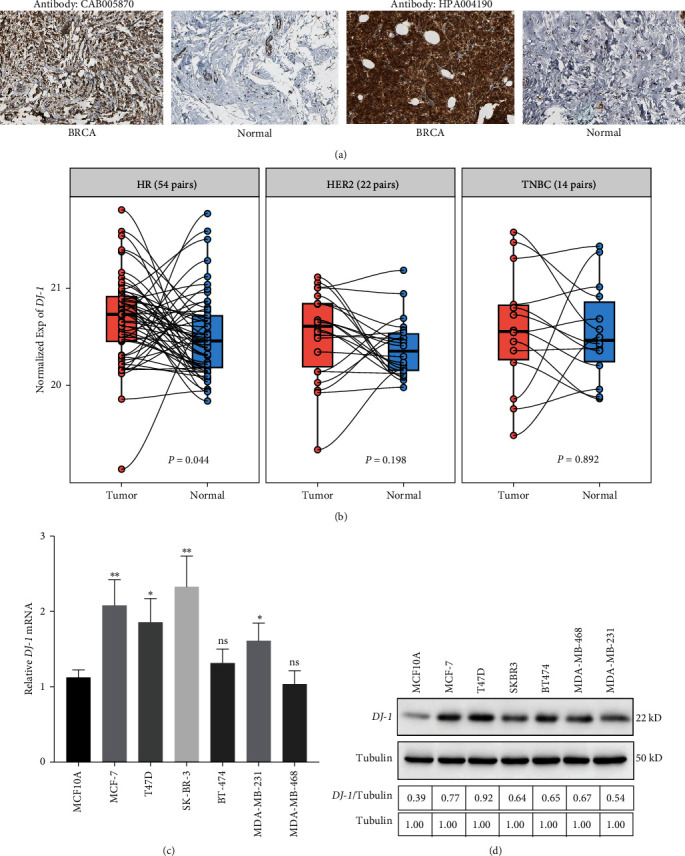
*DJ-1* expression in breast cancer tissues and cells: (a) the immunohistochemistry (IHC) images between breast tumor and normal tissues by two different *DJ-1* antibodies from the human protein atlas were downloaded by the Hpar packages in R; (b) differential expression of *DJ-1* in HR+, HER2+, or TNBC subtypes patients tumor samples compared to normal counterparts, respectively; (c) and (d) differential expression of *DJ-1* in nontumorigenic breast epithelial cell line (MCF-10A), HR+/HER2− breast cancer cells (MCF-7 and T-47D), HER2+ breast cancer cells (SK-BR-3 and BT-474) or TNBC cells (MDA-MB-231 and MDA-MB-468) by qPCR and western blot.

In order to explore the relationship between *DJ-1* expression and breast cancer patients, we analyzed the clinical characteristics of HR+ breast cancer patients (Table 1). The expression of *DJ-1* was significantly higher in tumor samples compared to that in adjacent normal counterparts, both in total breast cancer and HR+ subtypes patients, respectively (Figures 1(a) and 1(b), *P* = 1.29 × 10^−7^ in total patients and *P* = 7.64 × 10^−7^ in HR+ subtype). We then conducted a meta-analysis of *DJ-1* expression in the Oncomine database with criteria as *P* < 0.05, log_2_ fold change ≥1 and top 10% gene rank. We found that *DJ-1* was upregulated in all 11 analyses (Figure 1(c)). Interestingly, our analysis revealed that *DJ-1* was increased in the HR+ subtype in contrast to the HR-negative (HR−) subtype in both the TCGA database and METABRIC databases (Figures 1(d) and 1(e)). The expression of *DJ-1* was different in various subtypes of breast cancer (*Supplementary 2*). The immunohistochemistry results between breast tumor and normal tissues by two different *DJ-1* antibodies from The human protein atlas were also in accordance with its differential expression in tumor. Malignant cells generally displayed moderate to strong cytoplasmic and nuclear immunoreactivity (Figure 2(a)). The expression of *DJ-1* was significantly higher in HR+ subtypes patients tumor samples compared to normal counterparts but not in HER2+ or triple-negative breast cancer (TNBC, HR−, and HER2−) subtypes (Figure 2(b)). For validation, we performed *in vitro* assay. We verified the significantly high expression of *DJ-1* in HR+ breast cancer cells (MCF-7 and T-47D) compared to nontumorigenic breast epithelial cell line (MCF-10A), HER2+ breast cancer cells (SK-BR-3 and BT-474) or TNBC cells (MDA-MB-231 and MDA-MB-468) in mRNA and protein level via qRT-PCR and western blot assay (Figures 2(c) and 2(d)). All these results demonstrated that *DJ-1* was up-regulated in HR+ breast cancer.

Both in TCGA and METABRIC databases, PARK6, PARK9, PARK13, and PARK15 are positively correlated with *DJ-1*, while PARK8 is negatively correlated with *DJ-1*. Further research is needed to explore the role of other PARK family counterparts in HR+ breast cancer patients (*Supplementary 3*).

### 3.2. Association between DJ-1 and Clinic Features in HR+ Breast Cancer Patients

**Figure 3:**
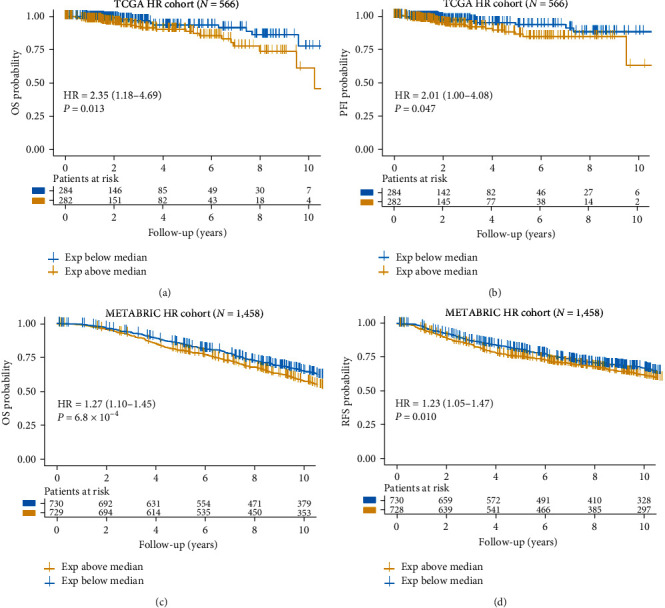
Association between *DJ-1* and clinical characteristics in HR+ breast cancer patients. (a) Kaplan–Meier overall survival curve of low and high *DJ-1* expression group in 566 patients from TCGA HR+ breast cancer cohort; (b) Kaplan–Meier progression-free interval curve of low and high *DJ-1* expression group in 566 patients from TCGA HR+ breast cancer cohort; (c) Kaplan–Meier overall survival curve of low and high *DJ-1* expression group in 1,458 patients from METABRIC HR+ breast cancer cohort; (d) Kaplan–Meier relapse-free survival curve of low and high *DJ-1* expression group in 1,458 patients from METABRIC HR+ breast cancer cohort.

Next, we explored the association between *DJ-1* expression and clinic survival in HR+ breast cancer patients. Patients with low *DJ-1* expression were found to have better OS and PFI/RFS in both the TCGA and METABRIC databases (Figure 3). This analysis showed that *DJ-1* expression could predict the prognosis in patients with HR+ breast cancer.

### 3.3. Association Analysis between DJ-1 Expression and the Levels of Infiltrating Immune Cells and Chemokines/Chemokine Receptors

**Figure 4:**
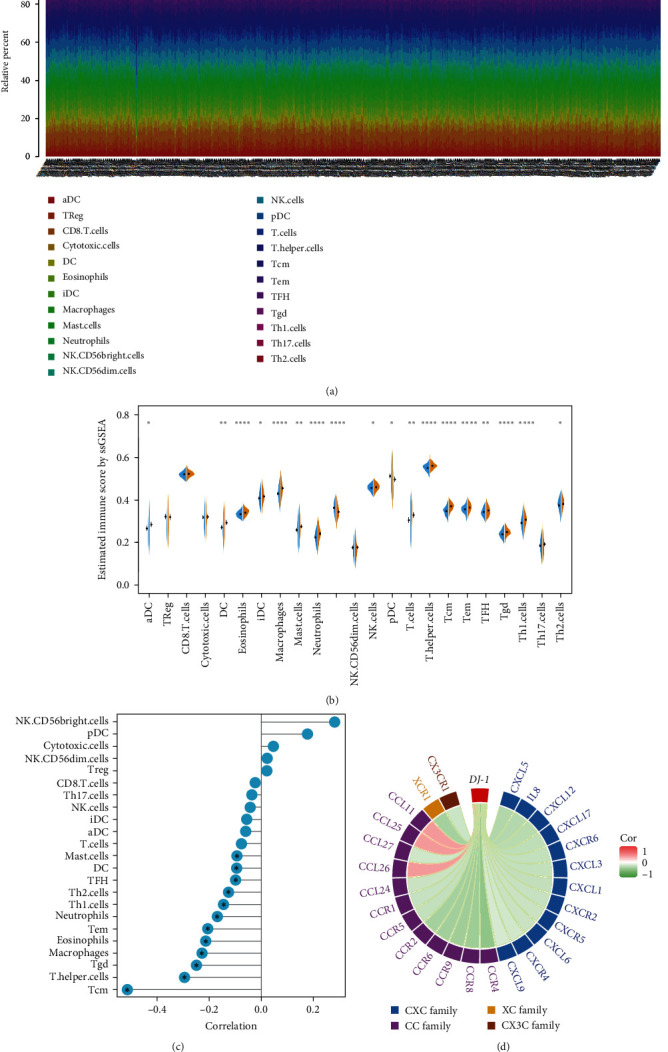
Association between *DJ-1* expression and the levels of infiltrating immune cells and chemokines/chemokine receptors: (a) heatmap of the levels of infiltrating immune cells; (b) comparison of the levels of infiltrating immune cells between the high and low *DJ-1* expression subgroups; (c) Tcm, T.helper cells, Tgd, macrophages, eosinophils, Tem, neutrophils, Th1.cells, Th2.cells, TFH, DC, mast cells were negatively associated with *DJ-1* expression in HR+ breast cancer; (d) correlation analysis of *DJ-1* expression with marker genes from chemokines/chemokine receptors in HR+ breast cancer.

Besides, we exhibited the landscape of *DJ-1* correlating with various infiltrating immune cells in HR+ breast cancers. *DJ-1* expression was negatively associated with Tcm, T.helper cells, Tgd, Macrophages, Eosinophils, Tem, Neutrophils, Th1.cells, Th2.cells, TFH, DC, MCs (Figure 4(a)–4(c)). To further explore the role of *DJ-1* in migration and immune cell function, we analyzed the correlation between its expression and chemokines and their receptors. The XC chemokine (XCR1), the CC chemokines (CCR1, CCR4, CCR8, CCR9, CCR6, CCR2, CCR5, CCL24, and CCL27), the CXC chemokines (CXCL5, IL8, CXCL12, CXCL17, CXCR6, CXCL3, CXCL1, CXCR2, CXCR5, CXCL6, CXCR4, and CXCL9) and the CX3C chemokine (CX3CR1) were downregulated when *DJ-1* expression level was increased. However, three chemokines, including CCL26, CCL25, and CCL11, were positively correlated with *DJ-1* expression (Figure 4(d)).

### 3.4. Association of DJ-1 with Mutational Landscape in HR+ Breast Cancer

**Figure 5:**
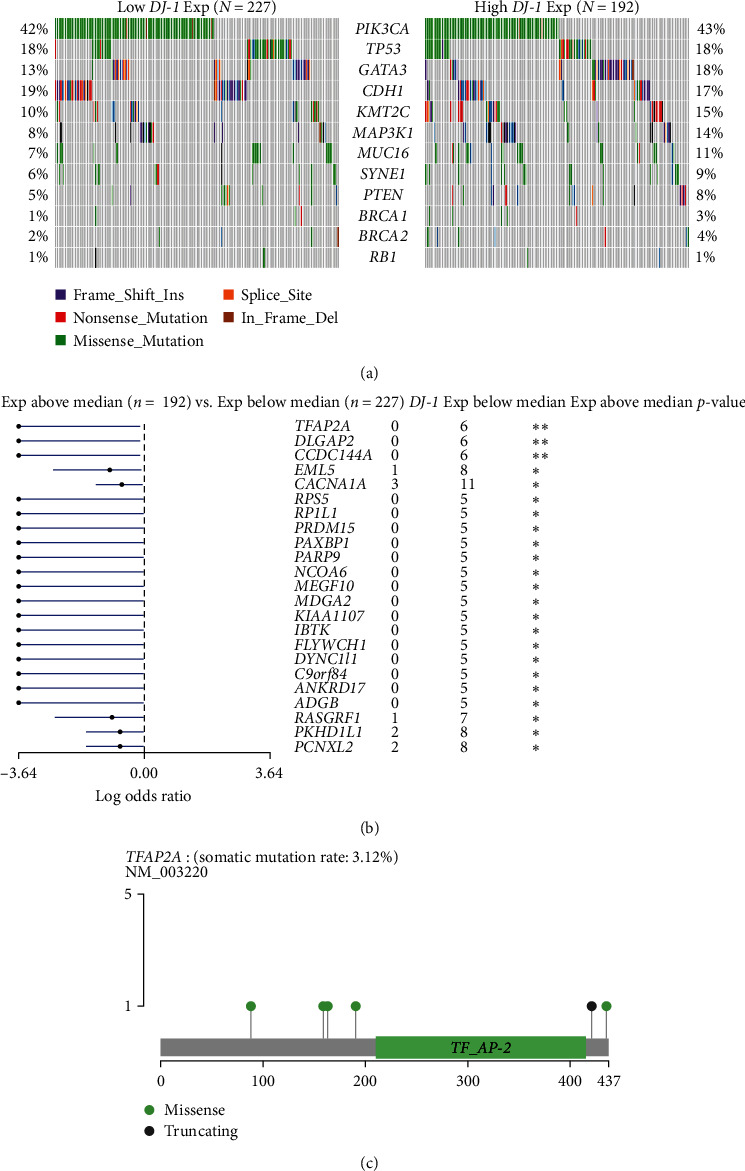
Association of *DJ-1* with mutational landscape in HR+ breast cancer; (a) the mutation rates of 12 frequently mutated genes between the high and low *DJ-1* expression subgroups; (b) the mutations in TFAP2A, DLGAP2, and CCDC144A were highly enriched in high *DJ-1* expression subpopulation; (c) TFAP2A mutation profile in high *DJ-1* expression subpopulation.

We evaluated the prevalence of somatic mutation in high and low *DJ-1* expression subpopulations.  Figure 5(a) shows 12 frequently mutated genes; APIK3CA, TP53, and GATA3 ranked the first three mutational genes.  Figure 5(b) compared the significantly different somatic mutations in *DJ-1* high and low expression subsets; mutations in TFAP2A, DLGAP2, and CCDC144A were most highly enriched in high *DJ-1* expression subpopulation. A total of six mutations in TFAP2A were detected, including five missense and one truncating (Figure 5(c)). The top 20 mutated genes in HR+ breast cancer, HER2+ breast cancer, and TNBC patients from the TCGA database were shown in *Supplementary 4*.

### 3.5. The Sensitivity in Immunotherapy and Chemotherapy

**Figure 6:**
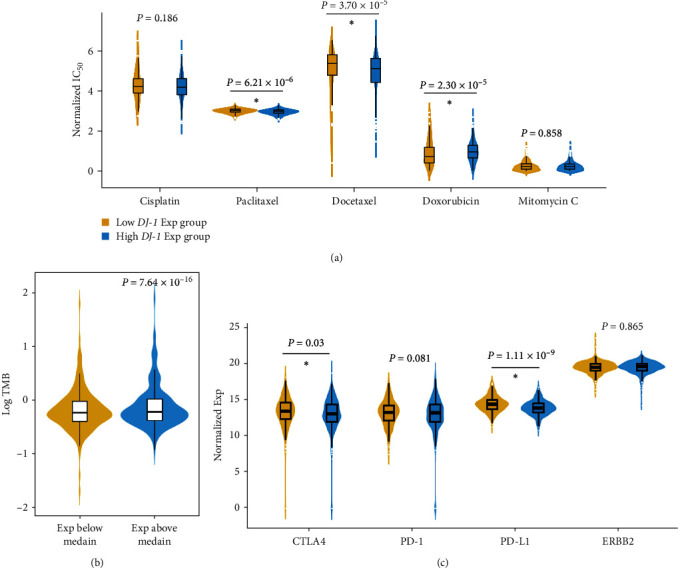
The sensitivity in immunotherapy and chemotherapy. The sensitivity in immunotherapy and chemotherapy between the high and low *DJ-1* expression groups: (a) the chemosensitivity to paclitaxel, docetaxel, and doxorubicin was found different between high and low *DJ-1* expression groups; (b) higher TMB was related with high level of *DJ-1*; (c) CTLA-4 and PD-L1 showed higher sensitivity in the high-*DJ-1* expression group. IC_50_: half maximal inhibitory concentration.

The clinical effects of breast cancer can be influenced by both drug chemosensitivity and drug resistance. Then, we analyzed clinical value in different *DJ-1* expressions via a ridge regression model. Paclitaxel and docetaxel showed more sensitivities in the low *DJ-1* group (*P* = 6.21 × 10^−6^ for paclitaxel and *P* = 3.70 × 10^−5^ for docetaxel) (Figure 6(a)). In contrast, doxorubicin was associated with higher sensitivity in the high *DJ-1* group (*P* = 2.30 × 10^−5^) (Figure 6(a)). We further observed high TMB levels in *DJ-1* high expression group (*P* = 7.64 × 10^−16^) (Figure 6(b)). CTLA4 and PD-L1, both known as two immunosuppressants commonly used in breast cancer, showed higher expression levels in *DJ-1* high expression group (*P*=0.03 for CTLA4 and *P* = 1.11 × 10^−9^ for PD-L1) (Figure 6(c)).

### 3.6. Functional Analyses

**Figure 7:**
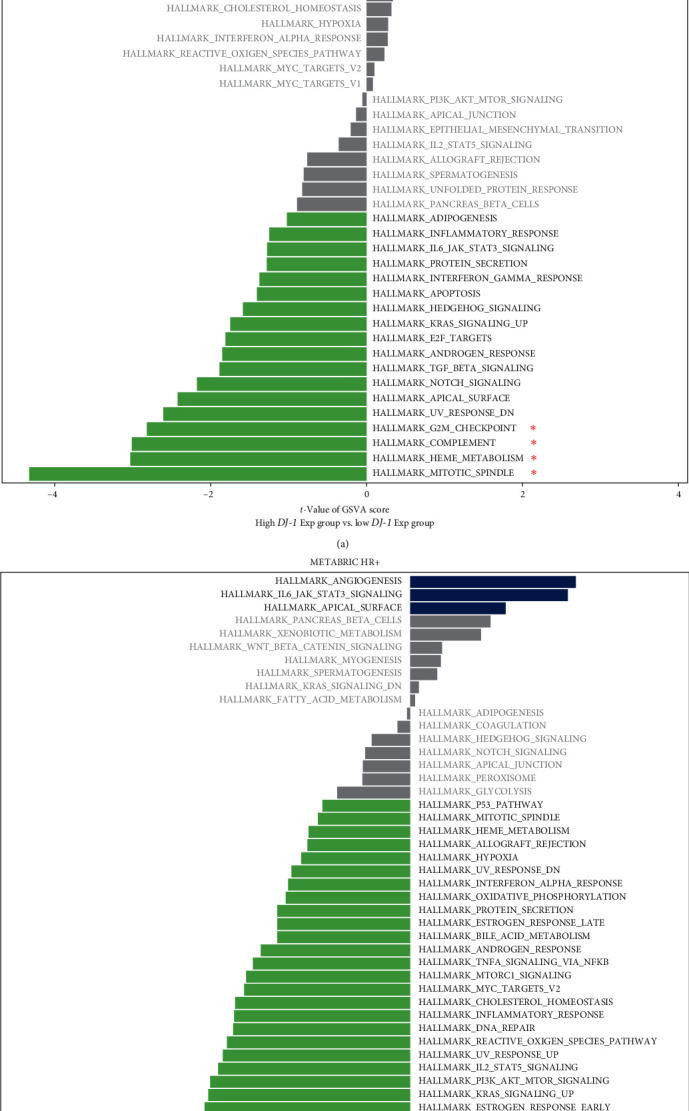
Pathway enrichment analysis by GSVA analysis in TCGA database (a) and METABRIC database (b).

The functional annotation of *DJ-1* in HR+ breast cancer was further explored. By the GSVA analysis, six pathways scores were found significant between high and low *DJ-1* expression groups in the TCGA database (Figure 7(a)), while nine pathways in the METABRIC database (Figure 7(b)). Taken together, two pathways, complement and G2M checkpoint were significantly activated in the *DJ-1* low-expressed subgroup in both TCGA and METABRIC database (*Supplementary 5*).

## 4. Discussion

*DJ-1*, a multifaceted protein that was first identified in Parkinson's disease, has been found with pleiotropic functions in multiple diseases ranging from neurodegeneration to ischemia-reperfusion injury [31, 32]. However, its role in breast cancer, especially in different subtypes, remains largely unknown. Thus, in this study, we profiled the expression of *DJ-1* in different breast cancer subtypes and found that *DJ-1* expression was evaluated in HR+ subtype. We mainly observed that high *DJ-1* expression group in HR+ subtype was associated with poor prognosis, low expression of chemokine receptor, high TMB, and more sensitivity to paclitaxel and docetaxel, highlighting the therapeutic potential and biomarker value of *DJ-1* in HR+ breast cancer subtype.

Recently, *DJ-1* exerted immune and inflammatory regulatory functions by regulating the activation of several immune cells, such as macrophages, MCs, and T cells, which has been supported by accumulating studies [26, 27]. However, there were few reports on the role of *DJ-1* in tumor immune microenvironment. In our research, *DJ-1* expression was negatively associated with deduced fractions of Tcm cells, TFH, DC, and MCs. Previous studies have found that Tcm cells produce higher levels of cytokines and have stronger cytotoxicity *in vitro*. In addition, Tcm cells had a longer survival time *in vivo*, showing a better ability to inhibit tumors [33]. Therefore, we suppose that the high expression of *DJ-1* might inhibit the invasion of Tcm cells in breast cancer and weaken its antitumor effect, thus promoting the progress of breast cancer.

In the tumor microenvironment, chemokines and chemokine receptors interacted to regulate the migration of a variety of immune cells into the tumor, thereby regulating the immune response in tumors [34]. T helper 1 (TH1) cells and natural killer (NK) cells had potent antitumor effects in the tumor microenvironment. CXCL9 and CXCL10 can recruited TH1 cells and NK cells into the tumors and played a role in tumor inhibition [35, 36]. In our study, the low expression of most of the chemokines, including CCL9 and CCL10 in *DJ-1* over-expressing HR+ breast cancer, might reduce tumor-infiltrating immune cells and suppressed antitumor immune responses.

The role of DLGAP2 and CCDC144A in malignant tumors is still unclear, but previous studies have suggested that TFAP2A can promote or inhibit cancer progression in tumors. TFAP2A, as a member of the AP-2 transcription factor family proteins, orchestrated a variety of cell processes, including cell growth, tissue differentiation, and apoptosis [37]. Many studies have shown that TFAP2A overexpression promotes the proliferation, migration, and invasion of breast cancer cells [38, 39]. However, the specific mechanism of TFAP2A in breast cancer remains unclear. The high mutation rate of TFAP2A may lead to the increased expression of *DJ-1* to promote the progression of breast cancer. Further research is needed to determine the specific mechanism.

The complement system is an important component of the inflammatory response in innate immunity and adaptive immunity. Complement proteins have an important role in the cognate interaction between antigen-presenting cells and T cells in immune response. For tumors, complement activation might be helpful in regulating T-cell response to tumors [40, 41]. The disruption of cell cycle checkpoints might allow cancer progression [42]. Sun et al. [43] found that activation of the G2/M cell cycle checkpoint might be resistance to CTL killing. Our study described a negative correlation between complement and G2M checkpoint and the *DJ-1* expression; *DJ-1* may be a key player in the inflammatory and immune responses.

Our results revealed that *DJ-1* might play different roles in different breast cancer subtypes, suggesting that *DJ-1* may be a specific marker for HR+ breast cancer, which providing a theoretical basis for further study of the heterogeneity between different subtypes. Moreover, *DJ-1* may become a predictive factor for precision treatment and immunotherapy of HR+ breast cancer. However, the specific functional role of *DJ-1* in the immunotherapy of HR+ breast cancer requires further in-depth experimental verification.

## 5. Conclusion

Overall, *DJ-1* was upregulated in HR+ breast cancer samples, and high *DJ-1* expression was associated with clinical prognosis, chemoresistance, and relevant immune features. Our findings indicated that *DJ-1* may act as a convincing prognostic marker and a predictor of therapeutic responses.

## Figures and Tables

**Table 1:** Characteristics of HR+ patients between *DJ-1* high and low groups in TCGA dataset.

Characteristics	Low expression group	High expression group	*N*	*χ* ^*2*^	*P*
Age (years)					
≤60	158	154	312	0.059	0.81
>60	138	140	278		
AJCC stage					
1	47	67	114	4.11	**4.26 × 10** ^**−2**^
2–4	243	226	469		
T stage					
T1	80	87	167	0.48	0.49
T2–4	216	207	423		
N stage					
N0	131	138	269	1.57	0.46
N1–3	162	151	313		
Not available^a^	3	6	9		
Pathological subtype					
Infiltrating ductal carcinoma	188	201	389	4.89	8.67 × 10^−2^
Infiltrating lobular carcinoma	84	62	146		
Other	24	32	56		
Menopause status^b^					
Pre	77	59	136	3.63	0.30
Peri	8	10	18		
Post	192	201	393		
Not available^a^	19	25	44		
Race					
Asian	9	17	26	18.84	**2.94 × 10** ^**−4**^
Black or African-American	22	52	74		
White	238	196	434		
Not available^a^	27	30	57		

^a^Data not available. ^b^Pre, <6 months since LMP AND no prior bilateral ovariectomy AND not on estrogen replacement, Peri: 6–12 months since last menstrual period; Post, prior bilateral ovariectomy OR >12 months since LMP with no prior hysterectomy. Bold values signify that *P* < 0.05.

## Data Availability

The datasets used and analyzed during the current study are publicly available in the TCGA (https://portal.gdc.cancer.gov/) and METABRIC (https://www.mercuriolab.umassmed.edu/metabric) databases.

## Supplementary Materials

Supplementary 1 Primers used for real-time PCR.

Supplementary 2 The expression of *DJ-1* was different in various subtypes of breast cancer.

Supplementary 3 Correlation between the expression of *DJ-1* and its family counterpart in TCGA cohort (A) and in METABRIC cohort (B).

Supplementary 4 Top 20 mutated genes in HR+ patients, HER2+ patients, and TNBC patients from TCGA database.

Supplementary 5 Complement and G2M checkpoints were significantly activated in the *DJ-1* low-expressed subgroup in both the TCGA database and METABRIC database.
