# Supplementary material for: DJ-1: A Potential Biomarker Related to Prognosis, Chemoresistance, and Expression of Microenvironmental Chemokine in HR-Positive Breast Cancer
Source: J Immunol Res. 2023 Dec 13;2023:5041223. doi: 10.1155/2023/5041223 (PMC10732869; doi:10.1155/2023/5041223)
Supplement: Supplementary 1 — Primers used for real-time PCR. [file 5041223.f1.pdf]

Supplement Table 1 : Primers used for real-time PCR.

Gene symbc	Forward primer (5′-3′)	Reverse primer (5′-3′)
DJ-1	GTAGCCGTGATGTGGTCATTT	CTGTGCGCCCAGATTACCT
β-actin	ATTGCCGACAGGATGCAGAA	GCTGATCCACATCTGCTGGAA
