# Supplementary figures and images for: DJ-1: A Potential Biomarker Related to Prognosis, Chemoresistance, and Expression of Microenvironmental Chemokine in HR-Positive Breast Cancer
Source: J Immunol Res. 2023 Dec 13;2023:5041223. doi: 10.1155/2023/5041223 (PMC10732869; doi:10.1155/2023/5041223)

A

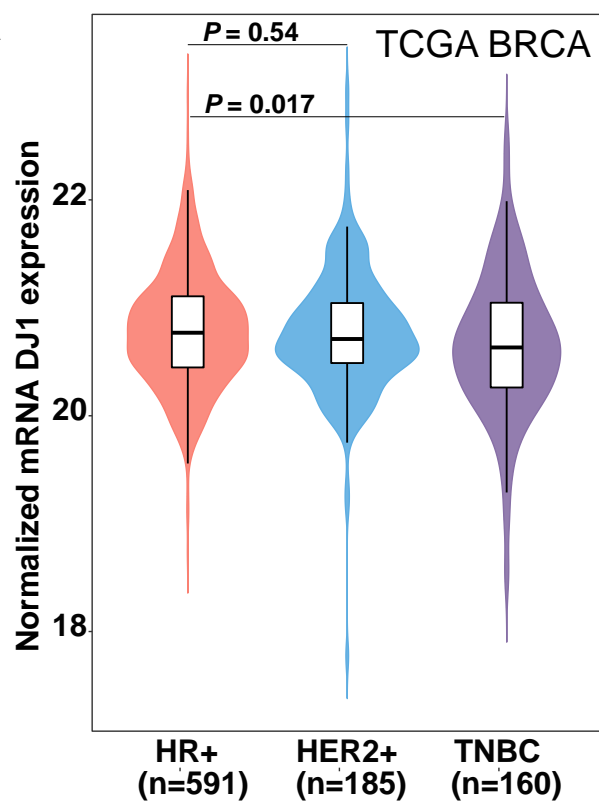

B

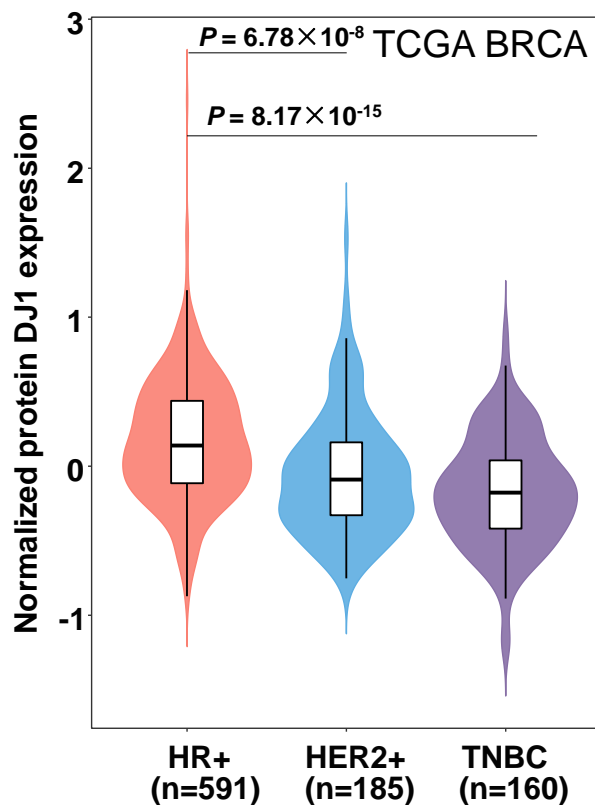

C

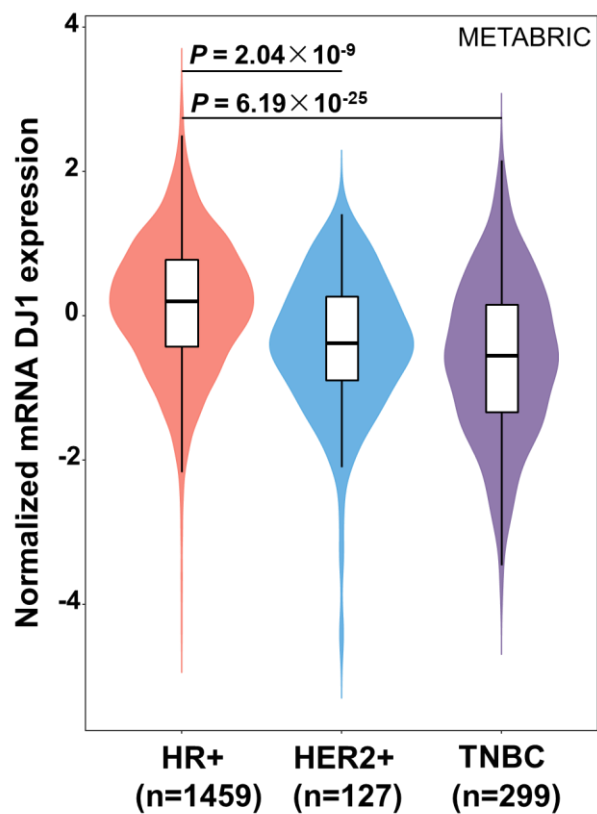

Supplement: Supplementary 2 — The expression of DJ-1 was different in various subtypes of breast cancer. [file 5041223.f2.pdf]

## PARK gene family in TCGA HR+ patients

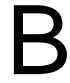

## PARK gene family in MetaBric HR+ patients

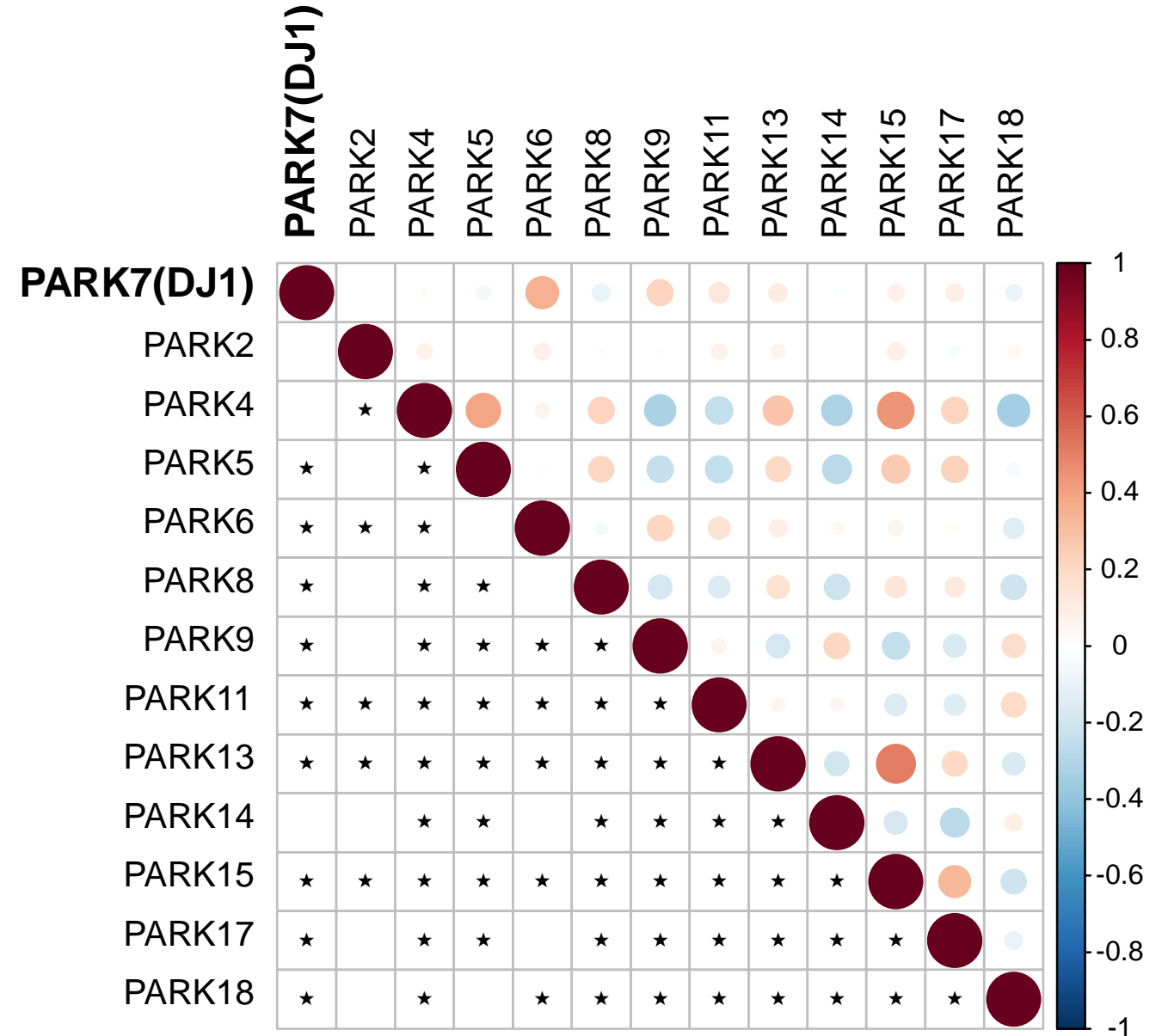

Supplement: Supplementary 3 — Correlation between the expression of DJ-1 and its family counterpart in TCGA cohort (A) and in METABRIC cohort (B). [file 5041223.f3.pdf]

TCGA

MetaBric

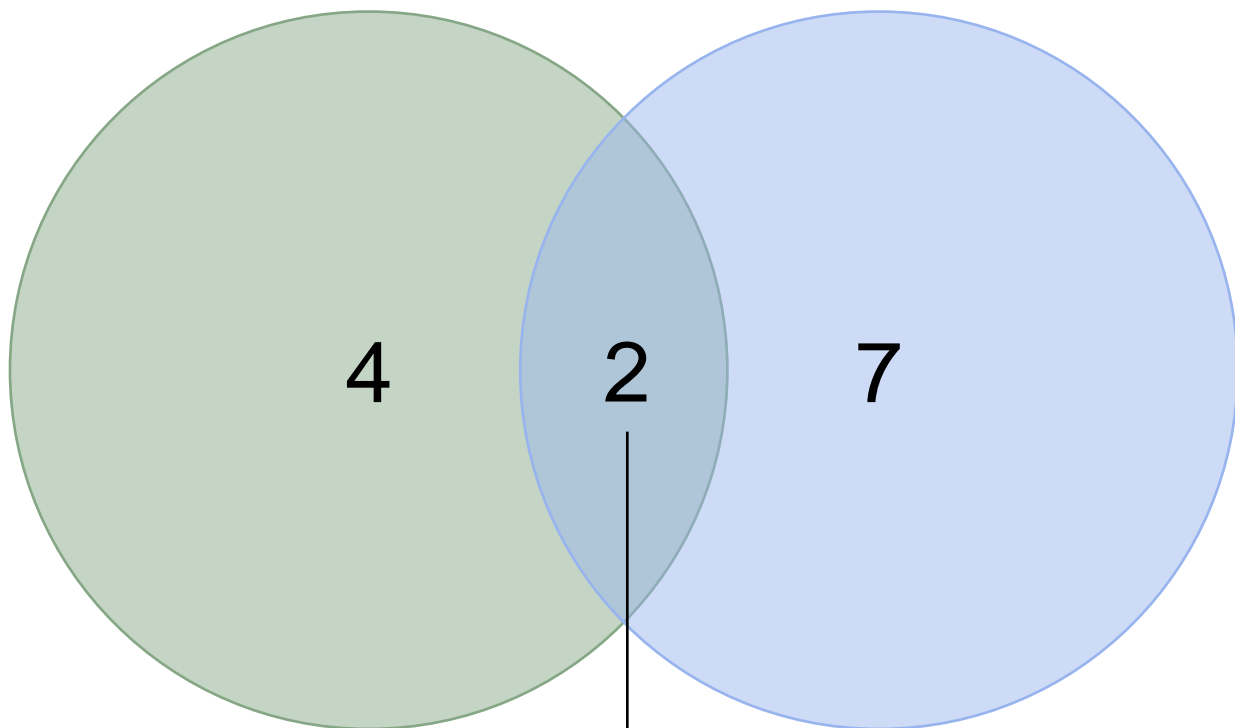

HALLMARK\_G2M\_CHECKPOINT

HALLMARK\_COMPLEMENT

Supplement: Supplementary 5 — Complement and G2M checkpoints were significantly activated in the DJ-1 low-expressed subgroup in both the TCGA database and METABRIC database. [file 5041223.f5.pdf]
