# Supplementary material for: DJ-1: A Potential Biomarker Related to Prognosis, Chemoresistance, and Expression of Microenvironmental Chemokine in HR-Positive Breast Cancer
Source: J Immunol Res. 2023 Dec 13;2023:5041223. doi: 10.1155/2023/5041223 (PMC10732869; doi:10.1155/2023/5041223)
Supplement: Supplementary 4 — Top 20 mutated genes in HR+ patients, HER2+ patients, and TNBC patients from TCGA database. [file 5041223.f4.pdf]

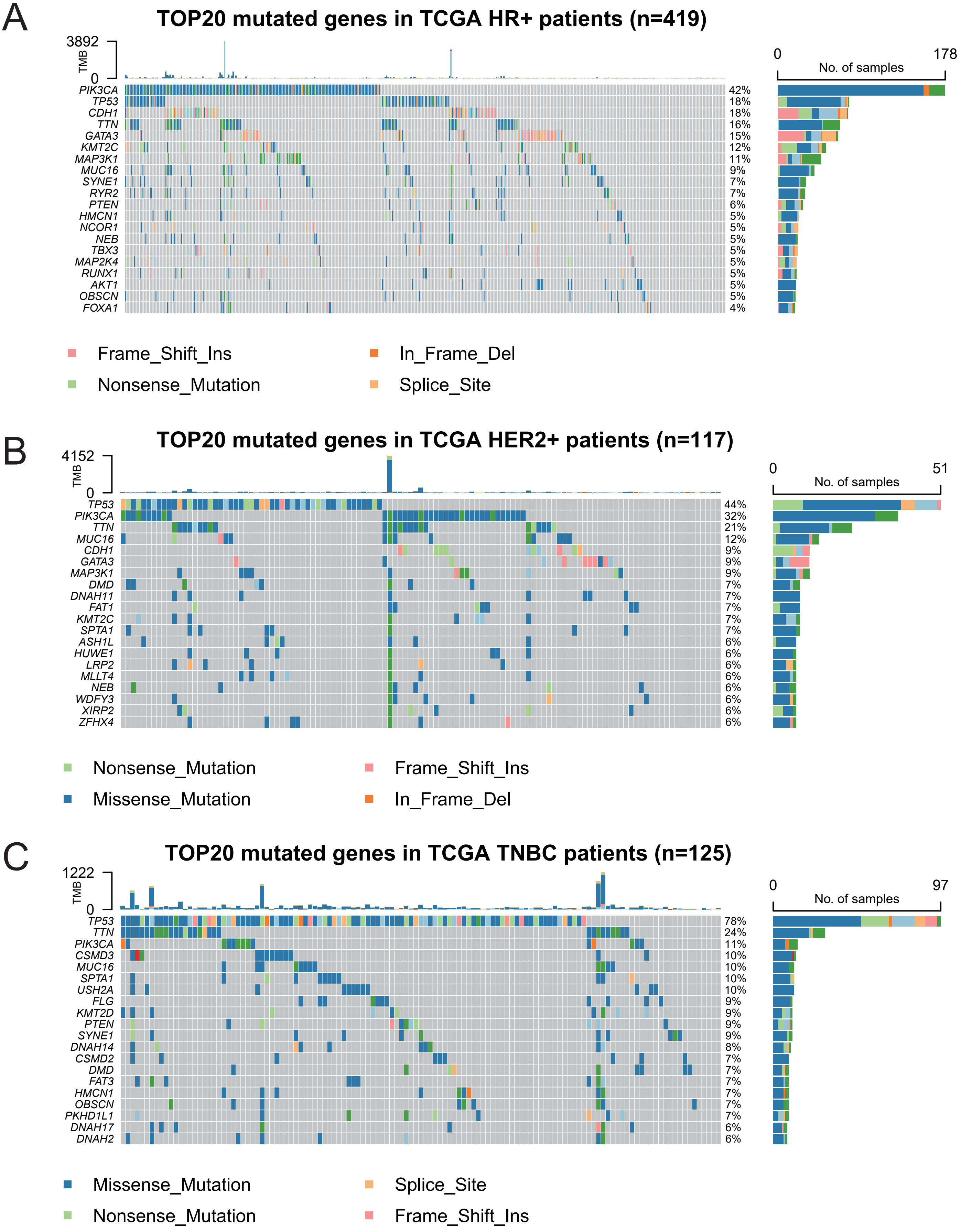

A
TOP20 mutated genes in TCGA HR+ patients (n=419)
3892
TMB
0
0
178
No. of samples
PIK3CA 42%
TP53 18%
CDH1 18%
TTN 16%
GATA3 15%
KMT2C 12%
MAP3K1 11%
MUC16 9%
SYNE1 7%
RYR2 7%
PTEN 6%
HMCN1 5%
NCOR1 5%
NEB 5%
TBX3 5%
MAP2K4 5%
RUNX1 5%
AKT1 5%
OBSCN 5%
FOXA1 4%
Frame_Shift_Ins
In_Frame_Del
Nonsense_Mutation
Splice_Site
B
TOP20 mutated genes in TCGA HER2+ patients (n=117)
4152
TMB
0
0
51
No. of samples
TP53 44%
PIK3CA 32%
TTN 21%
MUC16 12%
CDH1 9%
GATA3 9%
MAP3K1 9%
DMD 7%
DNAH11 7%
FAT1 7%
KMT2C 7%
SPTA1 7%
ASH1L 6%
HUWE1 6%
LRP2 6%
MLLT4 6%
NEB 6%
WDFY3 6%
XIRP2 6%
ZFHX4 6%
Nonsense_Mutation
Frame_Shift_Ins
Missense_Mutation
In_Frame_Del
C
TOP20 mutated genes in TCGA TNBC patients (n=125)
1222
TMB
0
0
97
No. of samples
TP53 78%
TTN 24%
PIK3CA 11%
CSMD3 10%
MUC16 10%
SPTA1 10%
USH2A 10%
FLG 9%
KMT2D 9%
PTEN 9%
SYNE1 9%
DNAH14 8%
CSMD2 7%
DMD 7%
FAT3 7%
HMCN1 7%
OBSCN 7%
PKHD1L1 7%
DNAH17 6%
DNAH2 6%
Missense_Mutation
Splice_Site
Nonsense_Mutation
Frame_Shift_Ins
